# Supplementary material for: National Assessment of Opportunities for Improvement in Preventable Trauma Deaths: A Mixed-Methods Study
Source: Healthcare (Basel). 2023 Aug 14;11(16):2291. doi: 10.3390/healthcare11162291 (PMC10454732; doi:10.3390/healthcare11162291)
Supplement: Supplementary file 1 [file healthcare-11-02291-s001.zip › healthcare-2491616-supplementary.pdf]

Code Number ____________________________

Preventable Trauma Death Pre-review & Panel Review Form

Pre-review Checklist

1 Dead?

☐ Yes ➔ **go to question #2** ☐ No (Not dead) ➔ **End of survey**

2 Is death due to trauma? **(Including due to sequelae or complication, regardless of death certificate, confirm with whole medical record)**

☐ Yes ➔ **go to question #3** ☐ No (Disease/Poisoning/Burn/Drowning etc.) ➔ **End of survey**

3 Basic information

■ Final Hospital ID: ____________________ ■ Sex: ☐ Male ☐ Female ■ Age : __________

■ Mechanism of injury: ______________________________ ☐ Blunt ☐ Penetrating ☐ Others

■ Time of injury: ____(YYMMDDHHMM)

■ Time of admission: ____(YYMMDDHHMM)

■ Time of death: ____(YYMMDDHHMM)

■ Certificates: ☐ Death certification ☐ Postmortem Examination ☐ none

■ Cause of death on 'death certification' (all causes as described): ____________________

■ Time from accident to death: (1) Within 1 hour (2) 1-6 hours (3) 6-24 hours (4) 1-7 days (5) 7-30 days (6) After 30 days

■ Location of accident and point

(1) Dead on Arrive (DOA) (2) Died at ER of Final hospital after CPR

(3) Died during operation (4) Died in ICU (5) Died in General ward

(6) Others ____

■ Admission department: ☐ General surgery ☐ Thoracic surgery ☐ Neurosurgery ☐ Orthopedics ☐ Emergency medicine ☐ Trauma surgery ☐ Others __________

■ Department where death occurred: ☐ General surgery ☐ Thoracic surgery ☐ Neurosurgery ☐ Orthopedics ☐ Emergency medicine ☐ Trauma surgery ☐ Others __________

4 Transfer

☐ Directly transported to a final hospital (Prehospital report: ☐ Yes ☐ No) ➔ **Go question #6 after #5**

☐ Inter-hospital transfer (number of times) ☐ Transferring hospital (address): ____________________

(Inter-hospital transfer Form: ☐ Yes ☐ No) ➔ **Go question #7 after #5**

5 Field Triage Decision Scheme (Duplicated checkable)

I. Step 1

(1) A/V/P/U: Below 'V' or GCS ≤ 13 (2) Systolic pressure < 90 mmHg (3) Respiratory rate < 10 or > 29

II. Step 2

Code Number ______________________________

(1) All penetrating injuries to head, neck, torso and extremities proximal to elbow and knee

(2) Flail chest (3) Two or more proximal long-bone fractures (4) Crushed, degloved, or mangled extremity

(5) Amputation proximal to wrist and ankle (6) Pelvic fracture (7) Open or depressed skull fractures (8) Paralysis

III. Step 3

(1) Falls - Adults: > 20 feet (one storey is equal to 10 feet)

- Children: >10 feet or two to three times the height of the child

(2) High-risk auto crash - Intrusion: >12 inches occupant site; >18 inches any site / Ejection / Death in same passenger compartment / Vehicle telemetry data consistent with high risk of injury

(3) Auto vs. pedestrian/bicyclist thrown, run over, or with significant (>20 mph) impact

(4) Motorcycle crash > 20 mph

IV. Step 4

(1) Age: > 55 or <15 (2) With significant burn injury

(3) End-stage renal disease requiring dialysis (4) Time-sensitive extremity injury

(5) Pregnancy > 20 weeks (6) EMS provider judgment

■ **Enough information to judge?** ☐ Yes ☐ No

■ **Source of information:** ☐ Pre-hospital report ☐ Medical records of hospital: ________ ☐ Others _______

6 Audit Filters for Prehospital Trauma Care

(1) Failure to secure appropriate airway

(2) Persistent hypoxia (SpO_2 < 90%)

(3) Failure to control a catastrophic external hemorrhage (compression or tourniquet)

(4) Field scene time >10 minutes (5) Field to hospital > 1 hour (Transport time:)

(6) Failure to get IV line

(7) Failure in Triage

(8) 기타 ______________________________

■ **Enough information to judge?** ☐ Yes ☐ No

7 Audit Filters for Interhospital Trauma Care

(1) Failure to secure an appropriate advanced airway before transfer

(2) malposition of an endotracheal tube

(3) Persistent hypoxia (SpO_2 < 90%)

(4) Failure to control a catastrophic external hemorrhage before transfer (compression or tourniquet)

(5) Transfer time > 1 hour (Time:)

(6) Inappropriate fluid (transfusion) resuscitation before departure or on the way of transfer

(7) Transfer to an inappropriate hospital

(8) Others ______________________________

Code Number ____________________________

■ **Enough information to judge?** ☐ Yes ☐ No

8 Audit Filters for In-hospital Trauma Care

(1) Timely response of required personnel and resources in attending to patient needs (e.g. response time of surgeons, availability of operating room);

(2) Operating room unavailable for the cases who need emergency operation

(3) ICU bed unavailable for the patients who need critical care

(4) Impossible to use specific equipment (mechanical ventilator, CRRT, rapid infusion system, CT...) or facility for the critical trauma cases

(5) Absence or lack of documentation for vital signs, GCS, or I/O while in emergency department

(6) Absence or lack of documentation for present illness, past history, and physical examination while in emergency department

(7) Glasgow Coma Scale score <13 and no head computerized tomography (CT) scan within 2 hours of arrival at hospital (if CT available in hospital);

(8) Glasgow Coma Scale score <8 and no endotracheal tube or surgical airway performed before leaving resuscitation area.

(9) Patient with abdominal injuries and hypotension (systolic BP <90) who does not undergo laparotomy within 1 hour of arrival at the hospital

(10) Abdominal, thoracic, vascular, or cranial surgery after 24 hours

(11) Unplanned return to operating theatre within 48 hours of initial procedure

(12) Transfusion after 15 minutes for definite hypotension due to traumatic hemorrhage

(13) Coagulopathy due to absence or delay of appropriate timely supply of coagulation factors

(14) Coagulopathy due to absence or delay in treatment for hypothermia

(15) Patients with >8 hours between arrival and debridement of an open fracture.

(16) Failure or delay of more than 5 minutes of airway management? Securement?

(17) Patient requiring re-intubation of the airway within 48 hours of extubation

(18) Non-fixation of femoral fracture in adult

(19) All delays in identification of injuries

(20) Hemothorax or pneumothorax due to absence or delay of timely chest tube insertion

(21) Failure of appropriate ventilation (SpO_2 < 90%, $PaCO_2$ > 60 mmHg, PaO_2 < 60 mmHg)

(22) Craniotomy after 4 hours, for drainage of epidural or subdural hematoma

(23) Delayed recognition of neurogenic or spinal shock, and absence or delay of early resuscitation with vasopressor infusion

(24) Other ____

■ **Enough information to judge?** ☐ Yes ☐ No

Code Number ____________________________

9 Summary and additional comments

____

____

➢ Pre-Reviewer Information

Pre-Reviewer

- ■ Review Date: yyyy / mm / dd ■ Reviewer Institute/Name/Signature:

Moderator

- ■ Review Date: yyyy / mm / dd ■ Reviewer Institute/Name/Signature:

Panel Review Checklist

I. Locations of the deficiencies (Duplicated checkable)

(1) Prehospital (2) Inter-hospital transfer (3) In-hospital

(4) Nothing (5) Others ____

II. Cause of death (Duplicated checkable)

(1) Bleeding (2) MODS / Sepsis (3) Respiratory arrest (4) Cardiac arrest (5) CNS (6) Others __________

III. Injury severity and quality of care

1. Severity of injury?

(1) Injuries and sequelae non-survivable even with optimal management

(2) Injuries and sequelae severe but survivable

(3) Injuries and sequelae considered survivable

2. Quality of Care?

(1) Evaluation and management appropriate according to accepted standards

(2) Some deviations from standard of care that may, directly or indirectly, have been implicated in patient's death: ____________________________________

(3) frank deviations from standard of care that, directly or indirectly, caused patient's death: ____________

(4) Refused treatment by patient or attorney

3. Co-morbid factors

☐ If patient had co-morbid factors these were major contributors to death: ________________

IV. Preventability

(1) Preventable (P) (2) Possibly or Potentially Preventable (PP)

(3) Non-Preventable (NP) (4) Non-Preventable, but with care that could have been improved (NPCI)

☐ Lack of information to judge: ____________________________

Code Number ______________________________

V. Summary and additional comments

➢ Reviewer Information

Designated panel ■ Subspecialty: ☐ GS ☐ CS ☐ NS ☐ EM ☐ 기타 _______________

■ Review Date: yyyy / mm / dd ■ Reviewer Institute / Name / Signature:

Panel team ■ ID : ■ Team leader:

■ Review Date: yyyy /mm / dd
